# PB.03: Evaluating the role of breast MRI at mid-cycle chemotherapy

**DOI:** 10.1186/bcr3505

**Published:** 2013-11-08

**Authors:** D Santosh, G Uruski, A Borley, P Young

**Affiliations:** 1 Cardiff and Vale University Health Board, Cardiff, UK; 2 Velindre NHS Trust, Cardiff, UK

## Introduction

The aim was to evaluate whether breast magnetic resonance imaging (MRI) following mid-cycle chemotherapy was influencing the neoadjuvant chemotherapy (NAC) regimen.

## Method

Thirty-two patients with breast malignancy receiving NAC were included in this retrospective study. Baseline and mid-cycle MRI were performed. NAC regimen and breast MRI reports were obtained from an online database. Tumour response was assessed by calculating percentage of volume reduction between interval scans. The response was categorised arbitrarily as good, moderate and poor with >50%, 20 to 50% and <20% reduction in tumour volumes respectively.

## Results

Nine patients had second tumours detected on the baseline MRI. The index tumours (*n *= 32) had showed good response in 23 (71.9%), moderate response in four (12.5%) and poor response in five (15.6%) on their mid-chemotherapy MRI. The five poor responders had good response in either the axillary node or in the second tumour. The second tumours (*n *= 9) showed good response in six (66.7%), moderate in two (22.2%) and poor in one (11.1%). No changes were made to chemotherapy regimen based on the MRI findings and these percentage tumour volume reductions.

## Conclusion

Our study shows that the percentage reduction in the tumour volumes demonstrated by the interval MRI scans are an important determinant of response to NAC. It is possible to obtain similar information from newer 4D sonography, which is cheaper and quicker to perform than MRI, and is better tolerated. In our practice, assessment at the mid-chemotherapy point is now undertaken with clinical assessment and ultrasound, which have provided adequate information regarding tumour responses to chemotherapy.

